# *Corynebacterium mucifaciens *in an immunocompetent patient with cavitary pneumonia

**DOI:** 10.1186/1471-2334-10-355

**Published:** 2010-12-17

**Authors:** Félix Djossou, Marie-Christine Bézian, Daniel Moynet, Anne Le Flèche-Matéos, Denis Malvy

**Affiliations:** 1 Division of Tropical Medicine and Imported Diseases, Department of Internal Medicine and Tropical Diseases, Hôpital St-André, University Hospital Center, 1 rue Jean-Burguet, Bordeaux, F-33075, France; 2 Department of Bacteriology, University Hospital Center, Bordeaux, F-33075, France; 3 Post-graduation sector in tropical medicine, University Victor Segalen Bordeaux2, 146 rue Léo-Saignat, Bordeaux, F-33074, France; 4 Laboratory for Urgent Response to Biological Threats, Institut Pasteur, 25 rue du Docteur-Roux, Paris Cedex 15, F-75724, France

## Abstract

**Background:**

*Corynebacterium mucifaciens *has been mainly isolated from skin, blood and from other normally-sterile body fluids. It has rarely been described as a human pathogen since its description.

**Case presentation:**

We herein report the first case of cavitary pneumonia due to *C. mucifaciens *in an immunocompetent man returning from Maghreb.

**Conclusion:**

*C. mucifaciens *should be considered as important human pathogen in patients with severe illness and compatible history of exposure even in individuals with no clearly identified immunosuppression.

## Background

Pathogenic manifestations of infection by *Corynebacterium *species have been mainly described to occur during *C. diphtheriae *infection, both among children or adult individuals [1]. Indeed, other strains belonging to the *Corynebacterium *group remain rarely recognized as human pathogens, mainly in immunocompromised patients [1-3]. To the best of our knowledge, we herein report the first case of a cavitary pneumonia associated with *C. mucifaciens *isolation from blood cultures made from an adult immunocompetent male patient.

## Case presentation

**Figure 1:**
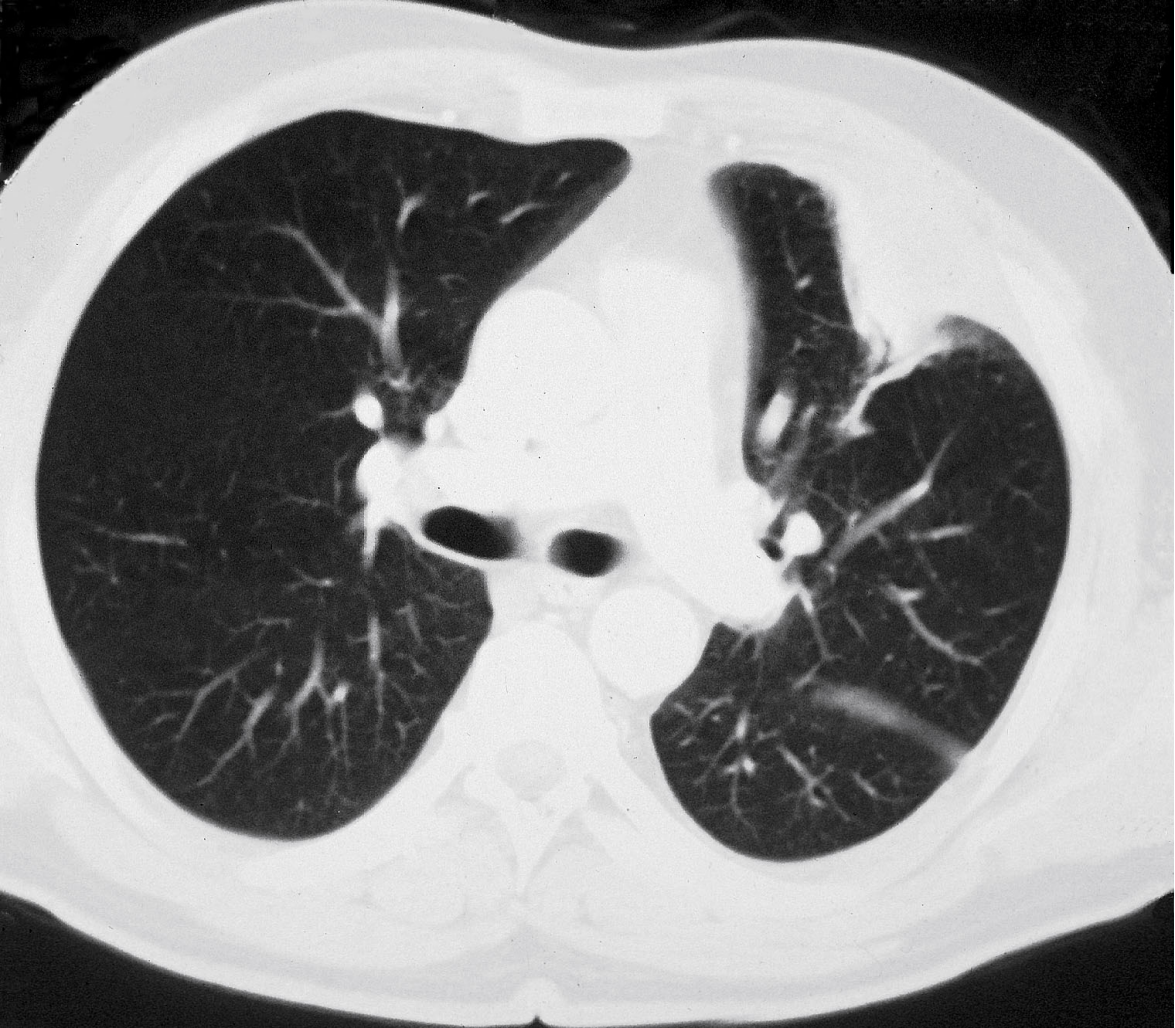
**Left-upper-lobe excavated infiltrate with pleural reaction in thoracic computerized axial tomography in a 50-year-old immunocompetent man**.

A 50-year-old man was referred to our clinic with a 10-day history of high-grade fever accompanied with severe dyspnea, dry cough and thoracic pain. He is Moroccan in origin and was living in France (Paris and then Bordeaux) for 20 years with a yearly 2- to 4-week stay in his country of origin. Indeed, he was visiting his family in Morocco for two weeks in a farm with regular exposure to horses and horse sheds when he became ill. At admission, the patient had general status impairment with a 12-kg emaciation, body temperature at 39.5°C and crepitant wheezes in the left hemi-thorax. Blood investigations showed a WBC count of 21 700/μL (neutrophils, 81%), an ESR of 111 mm/h (normal, <15), a glucose of 7.4 mmol/L (normal, 3.6-5.8), a fibrinogen of 10 g/L (normal, 2-4), a procalcitonin of 24 ng/mL (normal, <0.5) and a C-reactive protein of 280 mg/L (normal, <5). A chest roentgenogram and a thoracic computerized axial tomography revealed a left-upper-lobe excavated infiltrate with pleural effusion (Figure 1). Human immunodeficiency virus (HIV) infection was ruled out by serological testing and the patient was not immunocompromised (CD4+T cell count, 1355/μL). There was no recent or past history of alcohol intake. The only significative co-morbidity was a well-controlled type II diabetes mellitus. Other causes of defective immunity were also seeked, such as common immune variable deficiency, liver disease or malignancy.

**Figure 2:**
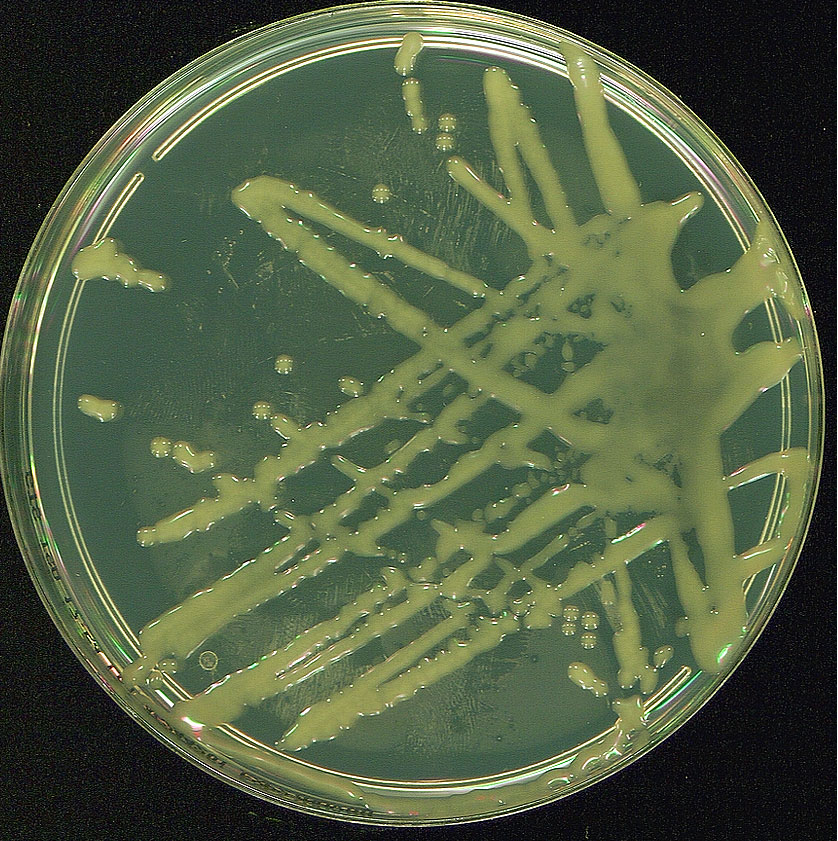
**Appearance of *Corynebacterium mucifaciens *colonies obtained in 24 h of incubation: circular, mucoïd, slightly yellowish and about 2 mm rods**.

Blood cultures were drawn twice and became positive after three days of incubation in an aerobic medium (Bactec plus aerobic/F-Becton Dickinson (BD) France) revealing diphteroïd, occasionally coccoïd non-motile gram-positive bacilli. The organism was sub-cultured on Columbia agar supplemented with 5% plain sheep blood (BD France) in aerobic and nutritive agar atmosphere at 37°C. Colonies produced in 24 h incubation were circular, mucoïd, slightly yellowish and about 2 mm (Figure 2). Firstly, physiologic tests realized in the API corynesystem (bioMérieux-F) strips were catalase positive, showed a positive reaction for pyrazinamidase, pyrolidonyl arylamidase, alkaline phosphatase, and acid production from glucose and maltose, were negative for oxydase, nitrate reduction, and urease. By this time, the attempt to identify the organism gave the numerical code 6100125 which corresponded, according to the system's database with the identification of *Corynebacterium striatum/amycolatum *with 88.7% similarity. Subsequently, a second API corynesystem was realized by the National Reference Centre for *Corynebacterium diphtheriae*, Institut Pasteur, Paris, France and gave the numerical code 6100104. The phenotypic identification was not changed but the acid production from maltose was negative like for the type strain of *C. mucifaciens *(GeneBank accession no. Y11200). Concurrently, the alternative diagnosis of *Rhodococcus equi *was not definitely ruled out considering both the mucoid colony morphology, the clinical presentation as cavitary pneumonia, and the recent direct exposure to premises used to house horses as consistent environmental reservoir of the latter organism.

**Figure 3:**
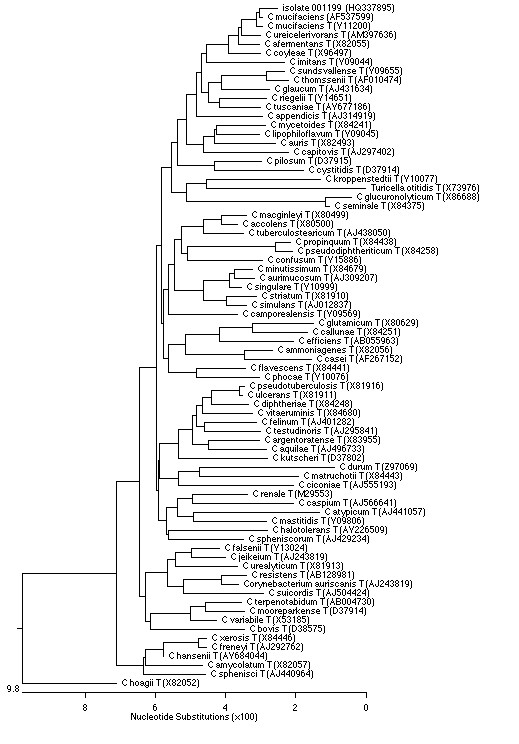
**Phylogenetic tree of 16 S rRNA gene sequences of isolate 001199 and representative *Corynebacterium *species, constructed by using the Neighbour-joining method**. Genbank accession numbers are given in parentheses.

On this behalf, the numerical code 001199 was given to the isolate and complete sequence analysis of the 16 S rRNA gene from the isolate was carried out as described previously [4,5]. A total of 1,208 continuous nucleotides were determined. Sequence of isolate 001199 (GenBank accession no. HQ337895) was compared to all bacterial sequences available from the GenBank database by using the BLAST program http://www.ncbi.nlm.nih.gov/blast/Blast.cgi and the MegAlign module of the Lasergene software (DNASTAR). A phylogenetic tree was generated by using the neighbour-joining algorithm [6] (Figure 3). Our isolate was found to 0.8% of divergence with the type strains of *C. mucifaciens*. Thus, isolate 001199 was definitely identified to belong to the species *Corynebacterium mucifaciens*.

Besides, the antimicrobial susceptibility pattern showed the strain was susceptible for β-lactams, amino-glycosides and glycopeptides. Treatment was initiated with rifampicin-spiramycin combination and was not changed even when the coryneform bacterium identification was later moved to *C. mucifaciens*. The patient rapidly improved in the general condition with clinical cure after four weeks but the treatment was continued for three additional weeks. Consistently, chest radiography after four weeks showed a minimal residual homolateral pleuritis involvement. At the end of the treatment, thoracic computerized tomography showed complete radiological recovery. The patient was followed during five years after the episode without relapse.

## Conclusions

Many new species of coryneform bacteria have been recently discovered and old species renamed, especially after molecular biology techniques were introduced. For many years, these organisms were disregarded as skin contaminants [7]. However, they have been recognized as important human pathogens, often acting as opportunistic pathogens in immunocompromised or severely-ill patients with symptoms compatible with bacteremia and without presence of other pathogenic organism [1-3,8]. Thus, eight strains of one of these new species had previously been isolated from human relevant clinical material. Concurrently, electron microscopy and comparative 16 S rRNA gene sequence analysis revealed that those formerly unknown coryneform bacteria belonged to a new subline within the genus corynebacterium and the name *C. mucifaciens sp*. was proposed [9]. Concerning the origin of the eight strains studied, they were isolated between 1992 and 1996. Of these, seven were from Switzerland and one from Germany. The clinical sources were six from blood, one from joint fluid and another one from wound swab. Clinical patterns associated with isolation were respectively intestinal bleeding, cardiac surgery, HIV infection, arthritis or cat bite, and for the three remaining, fever of unknown origin. No information was noticed concerning therapeutic regimen, outcome or risk factors for exposure to *C. mucifaciens *[9]. Concerning reports from human clinical specimens in non-European geographic areas, the evaluation of rare *Corynebacterium *species recovered in Canada have identified 23 strains of *C. mucifaciens *between 1985 and 2001. Of note, many of the clinical sources had been reported previously [9], with 10 from blood, three from abscesses or wound, although with the exception of recovery from dialysis and peritoneal fluid [3]. Detailed clinical information for underlying diseases of the patients was generally not available. More recently in the 2003-2005 period, five strains were identified in Japan from specimens of middle ear effusion cultures conducted in patients with otitis media with effusion, and four other strains were isolated from the nasal polyps and nasal discharge of patients with chronic sinusitis [10].

Although coryneform bacteria are commonly part of the normal flora of skin, their potential pathogenicity still remains to be assessed [1,2,7]. Indeed, these organisms have been increasingly implicated in serious infections and a fatal case of bacteremia due to an atypical strain of *C. mucifaciens *has been recently reported in an elderly Brazilian severely-ill woman [8]. Thereby, potential critical issue is stressing the importance for rapid and accurate laboratory identification and susceptibility testing of such unusual pathogens that might improve treatment and outcome of associated infection. In the case reported herein, the investigation was supported by a reference laboratory to confirm identification and provide molecular typing analysis of patient isolate.

With few exceptions, all *Corynebacterium *show good response to penicillin or vancomycin. In case of *C. mucifaciens*, beta-lactam antibiotics and aminoglycosides appear to have good activity [9].

From the sparse case series of patients infected with *C. mucifaciens*, no clear environmental exposure patterns have been recorded or evidenced. On the contrary, our patient had a recent history of contact with horses and horse sheds. Of interest, a study aiming to characterize the microbial exposure on farms using environmental dust has been previously conducted. This survey concluded that farms were highly exposed to different bacteria species including *C. mucifaciens*. Moreover, methods using single-strand conformation polymorphism were modified and validated for characterizing bacterial communities in environmental dusts. Results confirmed the transfer of microorganisms from animal-sheds (cow, chicken, and horse) to human environment [11].

In summary, the strain isolated in our case showed the most morphological and biochemical characters for *C. mucifaciens *identification: Gram staining revealing Gram-positive bacilli, circular, glistening mucoid and yellow colonies, consistent physiologic characters and molecular results, such as analysis of 16 S rRNA gene sequences. The strain identified was linked to a unique presentation of cavitary pneumonia that occurred in an immunocompetent man returning from Maghreb with horse contact and equine premises exposure, although the source of contamination and the transmission could not be formerly established.

## Competing interests

The authors declare that they have no competing interests.

## Authors' contributions

DMa, FD, DMo, AFM, MCB had full access to all of the data in the study and take full responsibility for the integrity of the data and the accuracy of the data analysis. DMa and FD have first seen the patient and had collected all information related to clinical and imaging data. DMa, MCB and AFM had conducted the analysis and interpretation of microbiological data. DMa drafted the manuscript. DMa, FD, DMo, AFM, MCB had critically revised the manuscript for important intellectual content. All the authors read and approved the final manuscript.

## Pre-publication history

The pre-publication history for this paper can be accessed here:

http://www.biomedcentral.com/1471-2334/10/355/prepub
